# Lived experiences of genetic diagnosis for rare disease patients: a qualitative interview study

**DOI:** 10.1186/s13023-024-03058-4

**Published:** 2024-02-14

**Authors:** Antonia Modelhart, Dominique Sturz, Lydia Kremslehner, Barbara Prainsack

**Affiliations:** 1 Department of Political Science, University of Vienna, Universitätsstraße 1, 1010 Vienna, Austria; 2 Ludwig Boltzmann Institute for Rare and Undiagnosed Diseases, Vienna, Austria; 3 Pro Rare Austria - Austrian Rare Disease Alliance, Vienna, Austria; 4 ePAG (European Patient Advocacy Group) ERN-Eye, Strasbourg, FR France; 5 Retina International Usher Syndrome Committee and Genetic Diagnosis Task Force, Dublin, Ireland; 6 Usher Initiative Austria, Vienna, Austria

**Keywords:** Rare diseases, Healthcare system, Genetic diagnosis, Patient experiences, Qualitative interview study

## Abstract

**Background:**

Genetic diagnosis is often understood as a single event within the care pathway of rare disease patients. Legal, policy and ethical scholarship focusing on rare diseases and genetic information discusses questions of how to best deal with the process of genetic diagnosis and the communication of genetic information within a given health system. We co-created a research design with rare disease patients and their families in Austria to explore in-depth the experiences of genetic diagnosis for people affected by rare diseases. Our objective was to trace the whole pathway of genetic testing and understand how rare disease patients experience genetic diagnosis as part of their care pathway in the healthcare system.

**Results:**

Data was collected through in-depth semi-structured qualitative interviews with 14 patients with a suspected or diagnosed rare disease or their parents, focusing on their perception of the pathway of genetic diagnosis in Austria. This pathway included the initial triggering of genetic diagnosis, the process of testing and its immediate (communication of results, counselling) and long-term, wider aftermath. Patients missed a clear link to already established forms of care such as their primary care/treating physicians. They also advocate for an integrated and interdisciplinary care pathway.

**Conclusions:**

Our study underscores the importance of a continuous care and communication pathway spanning from the initial genetic diagnosis process to post-test phases. It further shows the importance of exploring patients’ perspectives through qualitative research methods to understand the intricate workings of public health policies and tools. Integrating genetic diagnosis into a broader care trajectory is crucial for a holistic approach to care for rare disease patients who often rely on regular interactions with the healthcare system. Achieving this holistic approach requires collaboration between experts in specific rare disease areas, primary care physicians, and support networks.

## Introduction

**Fig. 1:**
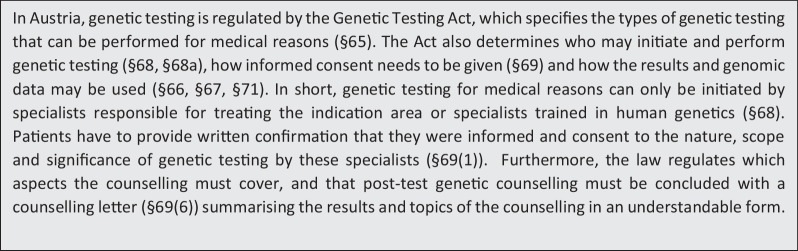
Regulatory framework for genetic diagnosis in Austria

A “rare disease” is one that affects no more than 5 persons in 10,000. To date, about 6,000 disease entities are considered rare according to this definition. In Austria, a country of just under nine million, over 400,000 people are estimated to live with a rare disease [1, 2]; across Europe this number is estimated to be about 30 million [2]. The advance of molecular technologies in recent decades has greatly increased possibilities for diagnosis [3, 4]. The European database for rare diseases, Orphanet, currently lists 6,172 diseases as unique rare diseases with 71,9% having an underlying genetic cause and 69,9% manifesting in childhood (ibid: 168). In this article, we explore how a patient cohort of 14 people living with 9 different rare diseases in Austria experienced the process of genetic diagnosis. We pay particular attention to how the experience of, and information obtained from, genetic diagnosis interacts with their own experience of the disease. Our interviews with patients (or, in cases of children, their parents) cover the entire genetic diagnosis process from the emergence of first symptoms to taking a DNA sample to discussing the genetic diagnosis results (Fig. 1 shows the regulations in Austria). For many rare disease patients, their disease histories are inevitably characterized by diagnostic uncertainty [5]. Against this backdrop, genetic diagnosis can serve as a material manifestation and proof of symptoms and open the door to therapy, participation in clinical trials, and other support infrastructures [4, 6]. It can also shed light on past and present experiences and reduce uncertainty through naming and framing what is experienced by rare disease patients and can end years of a diagnostic odyssey [6, 7].

Our aim in this paper is not to evaluate how well the deployment of clinical testing works in practice, but rather to gain a better understanding of how a number of rare disease patients experienced genetic diagnosis in the clinical setting. The focus therefore lies on the concrete practice of the genetic diagnosis as part of public health for rare disease patients. We therefore address several key aspects emerging out of our analysis: the uncertainties patients encounter within the diagnostic process, difficulties in communicating and transferring specific knowledge, but also the experiences of a scattered and fragmented patient journey.

While a rich literature relates to the technical implementation of genetic diagnosis in the context of rare diseases [8], there is also a limited body of work addressing societal and ethical aspects of genetic diagnosis for rare diseases (for a systematic review of ethical aspects see [9]). The questions addressed relate to informed consent, biobanking [10], secondary findings [11], parents of rare disease patients [12] and how rare disease patients experience the patient-doctor interaction [13], as well as understanding public attitudes towards genetic diagnosis [14]. For example, Etchegary et al. [14] found that in Canada, public interest in genomic sequencing is high but also public concern about privacy and critical attitudes about data use. In their analysis of rare disease patients’ views on knowing about secondary findings of whole-genome sequencing (WGS), Mackley et al. [11] found that patients had profound knowledge on the process of genetic diagnosis and relatively high levels of trust in healthcare professionals to decide which findings should be communicated to patients. Other works on rare diseases and genetic diagnosis discuss issues of uncertainty [15, 16] as everyday experience of biomedical encounters and rare diseases more broadly as well as the special role of patient organizations and patient activism in rare disease research [17, 18].

Although there are studies on patient understanding and satisfaction with genetic diagnosis [19] there is little qualitative research on patients’ experiences throughout the entire process of genetic diagnosis. For example, a study by Roberts et al. [19] shows high patient satisfaction with the communication but also a need to manage and temper patients’ expectation of the clinical relevance of such results, which are often high and not fulfilled. Research outputs often explore “attitudes” towards genetic research, whole genome sequencing and secondary or incidental findings [14, 20, 21]. We seek to add to existing literature an inductively oriented empirical account of how patients are experiencing the whole pathway, from the initial suggestion to undergo genetic diagnosis to the communication of results and the longer-term consequences.

A narrow understanding of genetic diagnosis as a tool and single event in the care pathway of rare disease patients falls short in considering the wider socio-medical relevance of genetic diagnosis. Hence, we put forward an approach to genetic diagnosis as a process for rare disease patients where communication of results, the transfer of knowledge, pre-test as well as post-test care are key parts as well.

## Materials and methods

### Research design

We opted for a collaborative research process that included rare disease patients as research partners. In a first phase (phase A, from January to May 2021), the first author of this paper (AM) put together a research team. After obtaining ethics approval by the University of Vienna (approval # 00606) AM recruited a research team in close contact with Pro Rare Austria, the Austrian Rare Diseases Alliance. AM contacted various patient organizations asking for potential research partners. Eleven representatives or group members from patient organizations were part of the initial research team. This team designed the specifics of the research plan and with the input of affected rare disease patients, meaningful and important dimensions could be considered. In phase A, the team met monthly to decide on approaches, methods, and the constructions of the interview guide (due to the pandemic, meetings took place online). We agreed on semi-structured qualitative interviews with rare disease patients in Austria. The interview guide aimed to explore four main categories: ethics, information, knowledge, and process. Using this guide, we examined each step, considered the consequences, and addressed ethical aspects. In phase B (from June to December 2021), 14 interviews were conducted, mostly via Zoom [10], two face-to-face [2], and one via telephone [1]. Interviewees were recruited through research partners and patient organizations as listed on the website of Pro Rare Austria. Interviews lasted between 30 and 60 min and every participant provided consent. All our interlocutors had undergone genetic diagnostics and most of them received a genetically confirmed diagnosis of a rare disease. In some cases, a rare disease was suspected but genetic diagnosis delivered a negative result. A negative result in this regard simply means that no known genetic variant associated with a disease could be found through testing [22]. Table 1 shows the interviewed patient cohort grouped for anonymization reasons in accordance with the European Research Networks.

**Table 1:** Overview of patient cohort

Rare diseases	n	Sex	Age
Rare neurological diseases	2	M	20–30
			30–50
Rare endocrinological diseases	1	F	30–50
Rare kidney diseases	1	M	30–50
Rare autoimmune diseases	3	F	30–50
Craniofacial anomalies and ear, nose, and throat disorders	2	F	20–30
			30–50
Connective tissue and musculoskeletal diseases	1	F	60 +
Not assignable	2	F	30–50
			50–60
Negative result	2	F	20–30
			30–50

### Data analysis

For all interviews, participant consent was obtained prior to the interview. All interviews were audio recorded and transcribed verbatim; names and other identifying details were removed from the transcript. Our interviews aimed at gaining an in-depth understanding of the complex subjective experiences of 14 rare disease patients to get to know varying positions in the context of genetic diagnosis. Data collection and data analysis took place in an interactive way: during the interview phase, the interviews were continuously coded and compared to derive key themes. For this, we used an approach inspired by Situational Analysis [23] as well as Constructed Grounded Theory [24] for mapping and coding the material. We read the interviews multiple times, wrote memos, and documented the discussions and interviews in a research diary. Employing an iterative process with MAXQDA (a software for qualitative data analysis), we initially coded the interview transcripts and then engaged in discussions. This iterative process allowed us to identify the main codes, which were discussed with our research partners during the workshops. Such codes included the diagnostic pathway and related codes such as family, knowledge, communication, management, counselling, consequences or more conceptual codes like uncertainty or temporality. Through analysis, we were able to abstract certain topics and locate it within wider issues of healthcare in Austria.

### Limitations of our study

Our research should be regarded in light of several limitations: First, our study took place in Austria and is therefore bound to the specifies of how genetic diagnosis is regulated by Austrian law and of the Austrian health care system. The aim of our study was not to quantify experiences along the pathway but to gain an understanding of the complexities of dealing with a rare disease and the process of genetic diagnosis. We had a small patient cohort (n = 14) and a limited variety of different types of rare diseases (shown in Table 1) and the focus on in-depth knowledge of experiences and subjectivities further limits the generalization of results. It is also highly important to point to the temporal dimensions of our research in such a dynamic field as genetic diagnosis: we reconstructed past events with our interlocutors, sometimes reaching as far back as 15 years. Thus, the experiences cannot represent or be considered generalizable to the up-to-date situation in Austria.

## Results

### The process of genetic diagnosis: how do patients get to genetic testing?

**Fig. 2:**
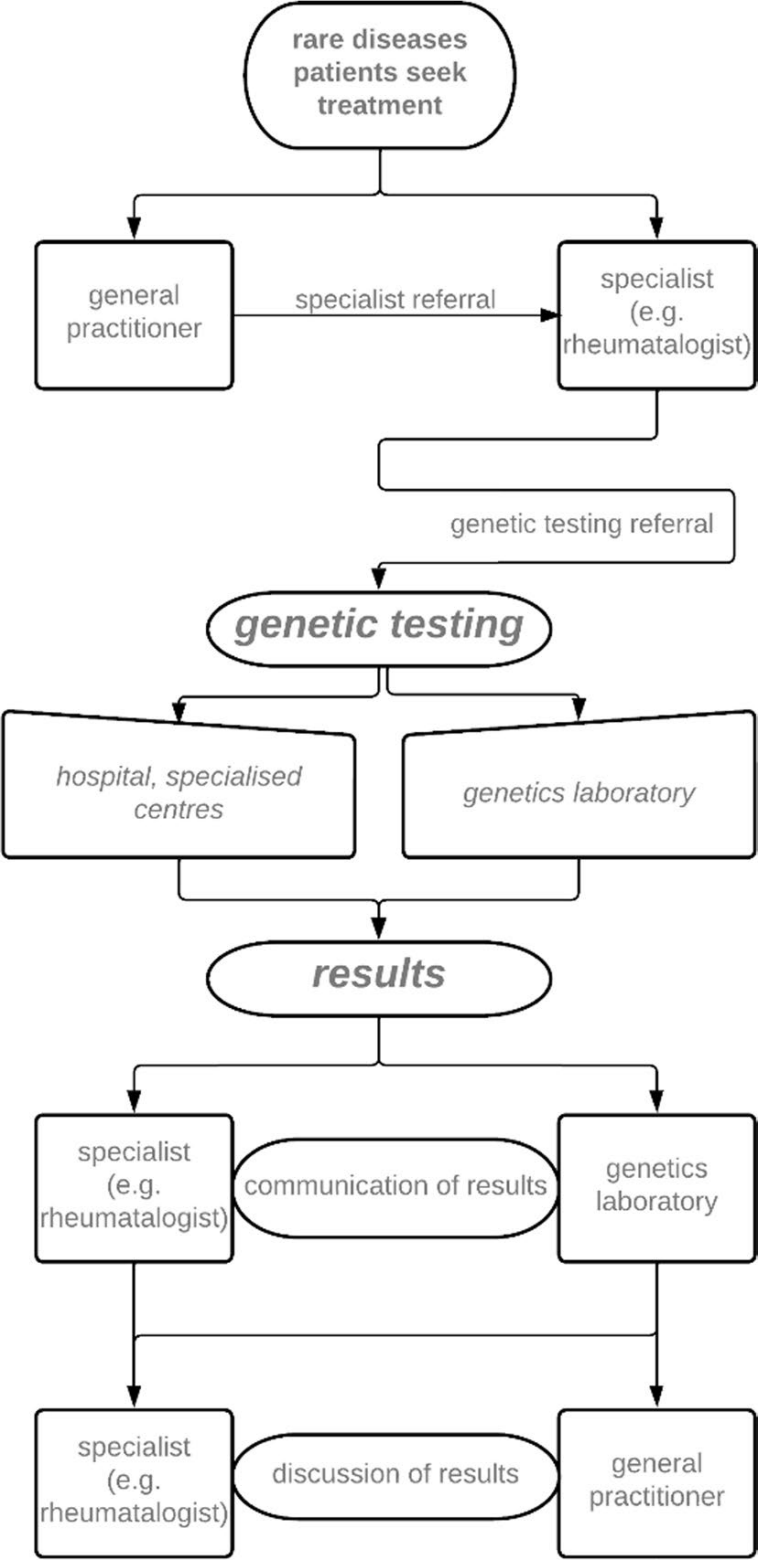
Genetic testing pathway in practice

As illustrated below (Fig. 2), in Austria, there are two main paths for patients to get tested, which in turn affects how patients experience the mediation of results. Patients either seek treatment by doctors specialized in the disease area most relevant for them (this need to pick one specialty when symptoms and phenotypic characteristics may require a more integrated approach is itself often problematic for rare disease patients). In this case genetic diagnosis takes place through the wider frame of medical disease management. For example, a patient with Lupus symptoms is assigned to rheumatology, and genetic diagnosis can be advised and proceeded from there on. When test results come in, the lab sends the results back to the doctor who requested the diagnosis, e.g. the rheumatologist, who then communicates the results to the patient. An alternative path would be for a patient to undergo genetic diagnosis directly via a human genetics laboratory, which then discloses the result to the patient directly. In some cases, patients chose a second opinion via a human genetics’ laboratory after they already had a genetic test as part of their disease management e.g. in a hospital. However, there are background infrastructures for rare diseases like the European Reference Network that are not in the picture presented in this paper. These are virtual networks connecting healthcare providers to facilitate discussions on complex cases but are mostly not visible and tangible for patients.

### From patients’ point of view

Most of the interviewees describe their way to genetic diagnosis as a journey of multiple years of uncertainty about their diagnosis and their disease, where genetic diagnosis provided at some point a glimpse of hope to get (a) certainty about health issues and (b) medical acknowledgement through a diagnosis that (c) points towards actionability. With actionability we refer to how the process of genetic diagnosis—with a confirmed diagnosis or a negative result—changed the way in which patients understood and went about various aspects of their lives. A diagnosis may guide action in the sense that it directly impacts how patients proceed with disease management. But it could also explain biographical disruptions and family histories, or it may be meaningful in that it helps other patients to make sense of their symptoms and experiences and thereby contribute to the rare disease community. One interviewee highlights the significance of shared information within databases and groups in their case. This approach helps them gain a better understanding of the disease and its potential course:

In many countries there are databases and groups where people meet each other, so you can make an approximate prognosis about the progression, but there is always a high degree of variability in it, you have to say that, depending on which exon or sometimes intron the mutation is located on. Apparently, there are also so-called modifiers, so there are modifier genes that could also have some influence on whether the progression is a little less severe or progresses very quickly, so there are many factors that have not yet been investigated in detail, but the mutation itself is an indication of the progression. (I14)

Our research participants entered the process of genetic diagnosis either because of a lack of diagnosis and diagnostic direction or for confirmation purposes. In the first case, genetic diagnosis is hoped to provide an explanation of health issues in situations of great ambiguity regarding the possible underlying disease. In the second case, health care professionals or patients themselves may have had a vague idea and genetic diagnosis was used to follow up on or genetic diagnosis is used to “prove” a diagnosis.

If patients lacked a diagnosis, the starting point for genetic diagnosis was a high level of uncertainty and often confusion that patients had experienced within the medical system. This uncertainty and confusion did not only pertain to the diagnosis itself, but also to the path of getting there. It was often not clear who is responsible and competent to care and treat for a wide range of symptoms. Rare disease patients faced the difficulty that the whole picture of their complex bodily symptoms did not map well onto a highly specialized medical system with expertise ordered according to specific diseases or organs. Many of the patients we interviewed had gone through years of stress and confusion: if doctors could not figure out a clear biomedical cause, the patient’s disease experience was often framed as being psychosomatic. Patients perceived such labelling as a dismissal of their experience and their suffering.

In the second situation, the starting point was a vague idea of a possible or presumed diagnosis on the side of patients and their doctors. Often it was the patients themselves who suggested a likely diagnosis or who initiated the process of genetic diagnosis:

Well, the history is a long one. I've had complaints since childhood, and over the decades, my GP has been suggesting that I might have something rare, needing a 'Dr. House,' so to speak. However, in the end, I was the one who suspected a diagnosis based on research and then initiated getting a genetic test. It wasn't the doctors' idea. (I6)

In the third situation, rare disease patients went for genetic diagnosis with the hope that it would confirm a presumed clinical diagnosis. In the following quote, for example, the patients’ clinical diagnosis of a rare disease was already known:

I went to [name removed for anonymization] hospital, they said they hadn't detected it yet. In the past, it could not be detected. Now it can. They recommended that our illness be diagnosed. Because when a medicine for us sick people comes on the market, you have to be able to prove that you have this disease. (I3)

This confirmation can be sought to be able to access therapeutic interventions or clinical trials, or to connect with people through patient organizations and support groups worldwide. It may help to find personal as well as medical acknowledgement or to set up patient organizations, and to process and cope with the diagnosis and its implications.

### Giving consent and giving blood

Our interviewees experienced the beginning of the genetic diagnosis process—the obtaining of a blood sample, the information and consent process—as unremarkable. It was insignificant throughout the process and memories of it were blurry. In some cases, patients could not clearly recall what they were told about the procedure of genetic diagnosis, e.g. that it requires a blood sample to be taken at first, and then a long amount of time to wait for the results. Further, patients only vaguely remembered the informed consent process and discussions about their data, the genetic information, and data protection. For some, it was just another test in a long history of medical testing. When asked about what patients could recall from the consent process, e.g. if data use was discussed at all or what kind of data is generated through genetic diagnosis, participants said that it was not a big issue and did not matter to them, as illustrated by this quote:

I believe that when it comes to medical issues, data protection stops with me. If sharing my data is the only chance I will have to access therapy at some point, then you can have my data—whatever you need. (I19)

But patient initiative and knowledge may also be required when genetic diagnosis was initiated by the treating specialist. Very often, treating physicians were unfamiliar with the genetic factors affecting a specific rare disease. This can have severe negative consequences for patients, as in the case of a young male with a neurological disease: Being diagnosed with a rare neurodegenerative disease through genetic diagnosis, his doctor gave a very poor prognosis of how the disease would develop over the next five to ten years. According to our interviewee, the doctor in charge did not consider or know of the many different factors that the prognosis depends on. Years after their fateful conversation with this doctor, our interviewee considered it a formative experience for them as a young adult:

The communication was extremely bad, so I think that's actually a scandal, what he said to me, he said, first of all, he didn't know his way around [this disease] at all, which is really weak for a neurologist, so I certainly know my way around better than he does, for example, he didn't know anything, he didn't know that I have these repeats, how often the genetic defect repeats itself on the DNA, so these repeats, the fewer they are, the weaker is the progression and my repeats are [number removed for anonymization] and at that time there were [number removed] and he should have said, yes, it is a bad diagnosis but with your repeats it is more or less within limits and he didn't know that. He just said that you will, as a matter of fact, be in a wheelchair in ten years at the latest, in five to ten years, you will lose the ability to walk, to speak, you will get a heart defect, so the worst things an 18-year-old can imagine. (I7).

For this patient, the traumatizing communication process and no formal post-diagnosis care led to years of denying the disease and avoiding medical encounters. Table 2 shows the extent to which it makes a difference to patients whether genetic diagnosis is provided by managing specialized physicians or by genetic counselling. Important dimensions such as the value of knowledge and the use of genetic diagnosis for patients vary, fulfilling different expectations and purposes. Patients can seek genetic diagnosis (1) in the context of a specialist or can turn (2) through referral by a specialist to a human genetic department.

**Table 2:** Overview of typical pathways in Austria

	(1) Genetic diagnosis through treating physicians (a specialist with specific knowledge in genetics)	(2) Genetic diagnosis through genetic counselling
Main points	Clear connection to treatment/disease management pathways, more established care relationships	Transparent and clear testing pathway
Value of knowledge	Diagnosis, proof, medical understanding, and orientation	Confirmation of a diagnosis made through other means, biographical explanation through family tree
Use for patients	Disease management, acknowledgement, post-test care	Explanation, knowledge, making sense, familiar consequences

A small number of our research participants experienced both pathways and were therefore in a position to compare their experiences along both of those paths: Patients going through genetic diagnosis via genetic counselling appreciated the clear procedure, the 30-min to 1-h long counselling explaining the results, its potential connection to other family members and the family history in general. But patients felt that it lacked direct connectivity to the medical treatment options, the possible therapies, and the dedicated research. If genetic diagnosis (option 1) had already taken place through a treating physician in the wider frame of disease management, patients more easily received advice on disease management and therapy options but missed a clear explanation of the scientific meaning of the genetic results.

### What happens after testing?

After results were communicated, a range of short and long-term consequences opened for patients: Is the result actionable, and if so, in what way? What does it mean in terms of treatment options? How to cope with a distressing diagnosis? How to communicate it to family members and friends? What are the implications for family planning? The result of genetic diagnosis was significant for patients also because it implicitly touched several dimensions: It affected patients’ relations to and understanding of their body, to the disease, to family members, and to the broader social world, as well as to the biomedical system and the potential community and networks surrounding a disease and can have lasting consequences.

For example, the patient’s own body, pain and symptoms were understood and reconfigured in the context of a diagnosis and thus a new (self-)understanding and relation to the body could be established. One interviewee emphasized the great relief of a diagnosis for making sense of their own illness biography:

I have found a picture for myself and I have even drawn it, a jigsaw puzzle, in my life I have had many individual parts, there was a diagnosis, there was a symptom complex, yes but, everything stood on its own, namely a heart disease is still there, everything on its own and through this genetically diagnosed disease one has found the frame of the jigsaw puzzle, normally one always starts with the frame of a jigsaw puzzle, that is the easiest, now we have the frame and then suddenly the individual parts or the individual partial pictures that were there, suddenly they find their place in this frame. (I6)

In another case, a patient experienced conflicting emotions of shock and relief after being diagnosed with a neurodegenerative disease. In the interview, the patient described that the diagnosis mitigated distress about their own psychic state:

But of course, it's a whole world that, I won't say collapses, but that is raised to another level because it also explained a lot about my psychological problems, etc. It all became clear to me that I'm not such an oddball because I only tell myself that, but that something was really wrong with me. (I19)

In other cases, patients received a negative result, indicating that genetic diagnosis could not detect a known or suspected disease-causing genetic variant. However, a negative result left the patients in ambiguous situations about the reason for a negative result: it may point to technological limitations, knowledge limitations (the variant is not yet known) or that the disease is not genetic, but no option can be ruled out. Some patients with a negative result were told to come again in a few years because of the dynamic developments in the field of genetic diagnosis:

I was told that it is very likely that it is some kind of connective tissue disease because a few tests were also carried out on my mobility, which is actually an indication of a connective tissue disease, but nothing could be proven and they said that perhaps there is some kind of connective tissue disease that is not yet known, but in two or three years they may already know more. (I1)

For patients, a negative result was difficult, but it may be especially problematic if the lack of a genetic explanation was taken by their physicians as an indication that they were not ill. Typically, the reasons for a negative result were not thoroughly considered, such as in the case of this patient:

They didn't find anything, and I was like “how? It can't be that you don't find anything in the genome”.—“Well, we did find something, we found a higher coverage of the chromosome set and requested extra tests” and (...) my problem is that they then said “Well, but in terms of your muscles you don't have anything” and I said “Like nothing, there's something, I mean, I'm not imagining that there's something”, well that's psychosomatic or something, these stories and then I was a bit angry, I have to say, because I say hey, I don't do ten years of diagnostics beforehand and don't do one test after the next and then they say there's nothing at all. (I8)

Our interviewees knew that something was wrong with them, and they felt that this should be acknowledged by the healthcare system even if they did not have a genetic diagnosis result to prove it. In such cases of failed medical encounters, most of our interviewees found very important comfort in patient organizations, networks, and online forums. Living in a small country made it even more difficult for patients with rare diseases to find physicians familiar with their specific condition. Many of our research participants had experienced stages through which their symptoms were dismissed as ‘psychosomatic’. This had dire consequences for them in terms of lack of treatment and acknowledgment and led to great frustration.

It was easier for patients to connect to patient organizations and rare disease communities through a positive result and a diagnosis. However, participants emphasized that patient organizations also actively discussed the lack of a diagnosis and opened a space to find some diagnosis through similarities, exchange of experiences and more without the official medical labelling.

Table 3 presents an overview of key themes that emerged from out interview data, ordered along the genetic diagnosis pathway (before, during and after genetic diagnosis). We also indicated the relation of these themes to broader societal issues. During the testing part of genetic diagnosis issues of unclear responsibilities and temporal references arose as well as confusion and frustration about the communication of results. After receiving the results, key themes revolve around short- and long-term consequences of having a positive or negative result, opening considerations about e.g. clinical but also scientific value.

**Table 3:** Overview of key themes along the pathway of genetic diagnosis

Steps	Key themes	Broad issues
***Before***		Genetic knowledge in society
*Reasons for testing*		
Lack of diagnosis and diagnostic direction	Uncertainty	
Vague idea of diagnosis	Medical acknowledgement	
Confirmation of diagnosis	Naming and framing	
*Expectations*		
Explanation		
Therapy		
Knowledge and acknowledgement		
***During***		Explanation of symptoms by “psychosomatic” factors
*Genetic testing*		
Process	Unclear responsibilities unclear timeframe Lot of expected personal initiative	
*Results*		
Mediation and setting	Lack of expertise in reading and mediating results	
***After***		Expertise and knowledge in health care
*Consequences*		
Short- and long-term	Directions for disease management and therapy Qualification for research projects/trials Orientation in health care system through diagnosis New self-understanding	

## Discussion

What lessons can be drawn from how rare disease patients experience genetic diagnosis in Austria? In our interview data, we identified several key issues that should be addressed. These issues concern the process of genetic diagnosis, and they also point to societal issues more broadly. For one, professionals could accommodate uncertainties through transparent and clear communication about the use, actionability, and meaning of genetic knowledge throughout the process. Second, because of the fragmented process, it was key for patients to have a clear picture about the responsibilities and steps along the pathway, which should be clear and easy to follow. Third, genetic diagnosis should be seen as a process rather than a time point, which means both patients and professionals understand diagnosis as a process that is not finished with a positive or negative result. The pathway continues in post-test care relations.

We will now discuss these issues in more detail and explain where our findings expand current scholarship. We will end with recommendations as to how these issues can be addressed.

Patients with rare diseases are used to dealing with high levels of uncertainty in medical encounters [15, 22, 25, 26]. They face challenges because of limited knowledge and treatment options, years of “the diagnostic odyssey” [29], lack of expertise or possibilities to connect to other patients due to the rareness of the diseases [5]. The literature is also specifically concerned with the role of uncertainty in exome and whole genome sequencing [7, 27, 28] pointing to the relationship of patients and doctors and the participation of patients in navigating ambiguous genetic diagnosis results. Stivers and Timmermans [7] argue that uncertainty in genetic diagnosis, rather than threatening the patient-doctor relationship can stabilize and configure a care relation. They show how geneticists handle diagnostic uncertainty through communicating and discussing ambiguity with patients, thus enacting engagement and care beyond a diagnosis.

As Rosell et al. [29] point out, even in case of a diagnosis through genetic means, for many rare disease patients, the “diagnostic odyssey does not necessarily end with a diagnosis”. For rare disease patients, a positive result often indicates that hardly anything is known or anyone else is affected. The authors found that even having a definite or likely diagnosis is often not as empowering but can isolate parents or patients: in the case of unique variants, not much is known, and it is hard to connect with other families. This very much corresponds with our results, where research participants experienced difficulties in finding other patients with the same symptoms or diagnosis to connect to, and with finding doctors experienced with their specific problems. Especially in a small country such as Austria, finding patients and families affected by the same disease was seen as particularly difficult. For many, the only way to connect to people with relevant knowledge is to interact with them online wherever they are in the world.

To address these variations of uncertainty in practice, we found that although genetic diagnosis offers diagnostic certainty in some cases, it often fails to prove or offer a diagnosis. Patients want clear communication about it right from the beginning. According to our data, in such a pre-genetic diagnosis phase it would be helpful for patients to know the following things as shown in Table 4.

**Table 4:** Relevant knowledge for patient in pre-testing phase

Process	Knowledge	Consequences
*What is being done?*	*What can be explained by genetic knowledge?*	*What outcomes or non-outcome spanning consequences are possible*
Patients want to know the steps of genetic diagnosis, the different phases and corresponding timeframes	Patients wish for a pragmatic evaluation of what a result may or may not indicate and what can be done about it in different versions	Patients want to be prepared for potential consequences for oneself, but also family members

Although, as noted, rare disease patients are accustomed to dealing with uncertainties when navigating the biomedical system, there are concrete issues in the process of genetic diagnosis that further intensify uncertainty. Many of our interviewees also suggested solutions. For example, for the problem of understanding genetic diseases and inheritance, they mentioned drawings as a supportive element to explain the basic idea of Mendelian diseases. In general, patients would benefit from a less dichotomized approach to genetic diagnosis results that goes beyond either having or not having a previously characterized genetic disease but considers nuances and scientific limitations: this means clinicians should not equate and treat a negative result as an indication that nothing is wrong. Clinicians should discuss with patients what a negative result might point to and how in light of a missing diagnosis, a caring relation can be maintained. Further, as Rabehariosa [22] argued, caring practices are also maintained through searching for a diagnosis. Similar to Latimer’s [30] notion of deferral, the “nuanced negative” (a negative result that is filled with uncertainty, potentiality and optimism) enables the maintenance of a caring relationship, to “keep looking for the answer” [22].

The participants in our study had to actively seek and maintain relations of medical care, run after their results, look for specialists etc. What was often missing for our participants was an active maintenance of caring practices on the side of their treating physicians. Most felt that they had to navigate the complex landscape of specialists and testing and treatment facilities on their own. In this context, patients mentioned the exceptional role that primary care physicians could play within the care pathway for rare disease patients: If rare disease patients had an intensive and trustful relationship with their primary care doctors, the biomedical care exceeds single steps and in the best case brings together various approaches to bodily problems. Patients appreciated the idea of having one central medical contact who is well informed about the experiences and characteristics of their specific rare disease, even if this figure was not a specialist for the disease itself. This constellation, patients stated, would help to explore and navigate the disease experience and disease management options better. Patients felt they could benefit a lot from a long-standing patient-doctor relationship: physicians’ knowledge can grow along that of rare disease patients’ experiences and knowledge, which creates both a trustful relationship and an epistemic acknowledgment of the rare disease patients’ specific experience and expertise. Primary care can pose an important instance for an integrated care approach that bundles and oversees the often necessary, multiple specialized treatments. To strengthen the role of primary care in the care pathway, according to our research participants, general practitioners should be more aware of considering a rare disease in searching for a diagnosis in the first place. It is impossible to know about every disease, but a broad awareness of rare diseases would help and should be part of medical education.

We argue that an important public understanding of genetic diagnosis should emphasize that it is part of a diagnostic process that does not end with a positive or negative result. As Patch and Middleton [31] point out, “the lifelong nature of genetic disorders, the fact that they may affect many different body systems requiring holistic coordination of care, and the fact that they may have intergenerational consequences and risks” (2020: n.p.) requires a nuanced understanding of genetic diagnosis, which highlights the relevance of a post-test care approach where the genetic diagnosis is just one part of an ongoing process of care.

Considering genetic diagnosis as a process and not as a bounded phase within the diagnostic pathway helps prevent several problems: For one, it helps to avoid treating a negative result as evidence of the absence of a disease. Further, due to evolving evidence and technological capabilities, our knowledge about the genetic cause of a disease is continuously increasing. These developments in genetic medicine help to identify more and more rare genetic diseases and shed light on yet undiagnosed diseases [3, 32]. Since 2010, close to 400 genes are associated to more than 800 “new” rare diseases [33].

Since knowledge and technical possibilities about rare genetic disorders increases, it is also important to keep in mind that genetic diagnosis at one point in time is not a finite answer or result. Finally, primary care can play a decisive role in accommodating uncertainties, offering a holistic approach to integrated care where rare disease patients are able to navigate disease management and therapy together with general practitioners.

## Conclusion

The experiences of rare disease patients provide valuable insights into the practice of genetic diagnosis, what works for them, and what needs improvement. Key issues identified here could inform the configuration of genetic diagnosis care pathways in Austria. Our structured qualitative evaluation of the experiences of rare disease patients with genetic diagnosis in Austria revealed that patients have a strong preference for a continuous care pathway that should be maintained from triggering genetic diagnosis to communication of test results and to the timely offering of available therapeutic options, if applicable.

Genetic diagnosis should be viewed as an ongoing process rather than merely a diagnostic tool that ends with communicating the results. This includes transparent and easy-to-follow discussions of what genetic diagnosis is, for example the different layers of a negative result. Post-test care should be an essential part of genetic diagnosis that is understood as a process as well. Right from the beginning of the process, patients and doctors should discuss hopes and expectations bound to genetic diagnosis, including what kind of vagueness and uncertainty it could hold. Integrated care—in accordance with primary care—can play a crucial role for a rare disease patient where the doctor-patient relationship can more easily be one of learning and navigating together on both ends. Primary care can be the interface of integrated care for rare disease patients that needs to attend to rareness. Thus, an “awareness of rareness” in primary care is key in establishing an often complex and multifaceted care pathway for rare disease patients. Focus should lie on integrated, as well as interdisciplinary care throughout and post-genetic diagnosis that binds together medical, social, and psycho-social aspects of rare diseases.

## Data Availability

The data that support the findings of this study are not publicly available due to their containing information that could compromise the privacy of research participants but are available from the corresponding author [AM] upon reasonable request.
